# Functionality of Bread and Beverage Added with *Brosimum alicastrum* Sw. Seed Flour on the Nutritional and Health Status of the Elderly

**DOI:** 10.3390/foods10081764

**Published:** 2021-07-30

**Authors:** Alejandra Rodríguez-Tadeo, Julio C. del Hierro-Ochoa, Jesús O. Moreno-Escamilla, Joaquín Rodrigo-García, Laura A. de la Rosa, Emilio Alvarez-Parrilla, José A. López-Díaz, María E. Vidaña-Gaytán, María N. González-Valles, Alfonso Larqué-Saavedra, Nina del Rocío Martínez-Ruiz

**Affiliations:** 1 Instituto de Ciencias Biomédicas, Universidad Autónoma de Ciudad Juárez, Anillo Envolvente del Pronaf y Estocolmo s/n, Ciudad Juárez C.P. 32310, Mexico; alrodrig@uacj.mx (A.R.-T.); jdelhier@uacj.mx (J.C.d.H.-O.); jesus.moreno@uacj.mx (J.O.M.-E.); jogarcia@uacj.mx (J.R.-G.); ldelaros@uacj.mx (L.A.d.l.R.); ealvarez@uacj.mx (E.A.-P.); joslopez@uacj.mx (J.A.L.-D.); 2 Instituto de Ciencias Sociales, Universidad Autónoma de Ciudad Juárez, Av. Universidad y Av. Heroico Colegio Militar s/n, Zona Chamizal, Ciudad Juárez C.P. 32300, Mexico; mvidana@uacj.mx (M.E.V.-G.); mgonzale@uacj.mx (M.N.G.-V.); 3 Unidad de Recursos Naturales, Centro de Investigación Científica de Yucatán, A.C. (CICY), Calle 43, No. 130×32 y 34, Chuburná de Hidalgo, Mérida C.P. 97205, Mexico; larque@cicy.mx

**Keywords:** elderly nutrition, functional food, *Brosimum alicastrum*, functional bread, functional beverage, Ramón seed flour

## Abstract

Physiological changes in elderly individuals (EI) can contribute to nutritional deterioration and comorbidities that reduce their quality of life. Factors such as diet can modulate some of these effects. The aim was to evaluate the functionality of foods added with *Brosimum alicastrum* Sw. seed flour in EI. EI (*n* = 23) living in nursing home conditions agreed to participate. A control stage was carried out (30 days) and subsequently, an intervention stage (30 days) was realized in which a muffin and a beverage, designed for EI, were added to the participants’ their usual diet. In both stages, anthropometric parameters, body composition, nutritional status, dietary intake, sarcopenic status, cognitive and affective states, biometric parameters, and total phenolic compounds (TPC), and antioxidant capacity in foods and plasma of EI were determined. The results showed that the consumption of the foods improved the energy intake and preserved the muscle reserves of the EI. The EI gained body weight (+1.1 kg), increased their protein (+18.6 g/day; 1.5 g/kg BW/day), dietary fiber (+13.4 g/day), iron (+4.4 mg/day), zinc (+1.8 mg/day), folic acid (+83.4 µg/day) consumption while reducing their cholesterol (−66 mg/day) and sodium (−319.5 mg/day) consumption. LDL-C lipoproteins reduced (14.8%) and urea (33.1%) and BUN (33.3%) increased. The TPC increased (7.8%) in the plasma, particularly in women (10.7%). The foods improve the EI nutritional status, and this has a cardiovascular protective effect that can benefit the health of the EI.

## 1. Introduction

The population of older adults is increasing rapidly, and projections indicate that by 2050 this group will constitute 30% of the world population. Physiological changes inexorably occur as age advances and with it a marked deterioration in those aged 70 years or above. The changes that accompany aging frequently present comorbidities that modulate and complicate any type of intervention. The aging process produces a series of changes, some reversible, especially in relation to body composition, physical activity, and dietary intake. Epidemiological studies show weight loss and a reduction in caloric intake as priorities at this stage of life [1]. In elderly individuals (EI) energy expenditure frequently exceeds energy intake, affecting the body weight (BW), muscle wasting, and increased weakness. These conditions provoke a decrease in muscle mass, metabolic rate, appetite, physical activity, among other changes, that affect the functional ability and health status of EI [2]. Aging causes anabolic resistance that limits the use of protein in muscle synthesis and protein requirements are thus recommended above the classic recommendations of 0.8 g/kg BW/day and that can reach 1.2 to 1.5 g/kg BW/day [3]. Muscle losses in 1% and 2% can occur in 1% and 2% over people from the age of 50 and loss of strength is directly connected with the reduction in muscle mass [4]. This involuntary loss of muscle mass, named sarcopenia, is part of the frail elderly syndrome and constitutes one of the main risk factors for disability and mortality in IE. Other changes in body composition such as the increase in fat mass have been associated with complications of other comorbidities in EI such as type 2 diabetes mellitus, hyperlipidemias, arterial hypertension, among others [5]. Therefore, diet is an important factor that can be modified to improve nutritional status and reduce the risk of common age-related diseases [6]. The development of new foods attending to the physiological changes of the EI could help to maintain a more active and healthy aging process. In the design of these foods, it is to address nutritional deficiency, functional ability and improve linking, among other factors [2]. Additionally, the incorporation of functional foods in the diet in combination with healthy habits and lifestyles can help promote healthy digestion, immunity, locomotion, and cardiovascular health, among others, in the elderly [7]. According to aging changes, we previously designed two foods for the elderly, a bread (muffin) and a beverage (“atole”), which were combined with *Brosimum alicastrum* Sw. seed flour (Ramón). *Brosimum alicastrum* Sw. is an endemic tree of the Mexican tropic whose underutilized seed has a high nutritional value [8,9,10,11]. The foods were designed considering a particularly high contribution of protein, dietary fiber, and micronutrients. These foods are gluten- and lactose-free, with a reduced level of caffeine and sugars. Therefore, the aim of the present study was to evaluate the functionality of the foods combined with *Brosimum alicastrum* Sw. seed flour on the nutritional and health status of the elderly.

## 2. Materials and Methods

### 2.1. Materials for Foods

The Ramón seed flour (RSF) (*B. alicastrum* Sw.) was provided by CICY (Herbarium Roger Orellana, Centro de Investigación Científica de Yucatán A.C.). The seeds were collected from growing Ramón trees at rancho Xoccheila (20°33′ N; 89°34′ W), in the municipality of Sacalum, Yucatán. The seeds were dried in the sun, the testa was removed, and the seed was ground to a fine flour. Other materials used for the foods were rice protein, fiber oak, and cocoa (Livenka**^®^**, Jiutepec, Morelos, Mexico), soy protein (GNC**^®^**, CDMX, Mexico), oat almond flour (Mandelin**^®^**, San Luis Obispo, CA, USA) corn flour (Maseca**^®^**, Gruma, Chihuahua, Chihuahua, Mexico), hydrolyzed unflavored whey protein (Muscle Feast**^®^**, Nashport, OH, USA), unsweetened almond drink d (Nature’s Heart**^®^**, Terrafertil**^®^**, Tultitlán de Mariano Escobedo, Estado de México, Mexico), unflavored whey protein isolate (Isopure**^®^**, GNC**^®^**, CDMX, Mexico), orange and cappuccino flavorings (Deiman**^®^**, Monterrey, Nuevo León, Mexico) and other materials such as egg, vanilla extract, baking yeast, baking powder, soy oil, non-caloric sweetened dried cranberries and muffin liners were purchased at the local markets of Ciudad Juárez, Chihuahua, México.

### 2.2. Bread and Beverage for Elderly

The foods were designed ensuring that they were products of smooth consistency with a significant contribution of protein and dietary fiber. Therefore, physicochemical characteristics of RSF were considered according to reported data by Subiria et al. [11]. The final formulation of the muffin was reached, with RSF in 43% of the flours. In addition, the product was combined with corn flour, rice flour, almond flour, rice protein, soy protein, hydrolyzed whey protein, oat fiber, egg, soy oil, cocoa, almond drink, vanilla extract, baking yeast, baking powder, non-caloric sweetener, dried cranberries, and orange flavoring. On other hand, a beverage (“atole”) was designed containing 9% of RSF and other ingredients such as whey protein isolate, oak fiber, cocoa, unsweetened almond drink, vanilla extract, non-caloric sweetener, purified water, and cappuccino flavoring were added. Physicochemical analysis and minerals (copper, potassium, iron, zinc, and sodium) of the foods were determined in triplicate, following the AOAC methods: moisture by oven method at 105 °C for 8 h (VWR**^®^**, Model 1324, Irving, TX, USA); ash in a muffle furnace (Felisa**^®^**, Model FE-340, Guadalajara, Jalisco, Mexico) at 550 °C for 5 h; crude protein by Kjeldahl method (Labconco**^®^**, Model RapidStill II, Kansas City, MO, USA) using as nitrogen to protein conversion factor of 6.25 for RFS and beverage, and 5.83 for muffin [12]; fat by Soxhlet method (Soxtec™, Model 2043, Foss™, Hilleroed, Denmark); total carbohydrates by difference method; dietary fiber by enzymatic-gravimetric assay, water activity in AQUALAB**^®^** (Model Serie 3, Meter Food, Washington, DC, USA) equipment, pH by potentiometric method (Accumet**^®^**, Model AB15 Plus, Westford, MA, USA) and minerals such as copper, potassium, iron, and zinc were determined by atomic absorption method and sodium by atomic emission method (Perkin Elmer**^®^**, Model AAnalyst 200 Spectrometer, Boston, MA, USA) [13]. Microbiological quality in total coliforms bacteria (CC), aerobics mesophilic bacteria (AC), yeast and mold (YM) were determined in both foods according to the plate count method (3M™, Petrifilm™, Minneapolis, MN, USA). Briefly, food samples were diluted 1:10 in saline solution (0.9%), homogenized and 1 mL was plated onto Petrifilm™. Plates were incubated at 35 ± 1 °C for 24 h for CC, 48 h for AC, and 25 ± 1 °C for YM. Official Mexican regulations were observed for the limits of CC, AC, and YM in the bread and the beverage [14,15].

Furthermore, a consumer acceptance test was carried out for the muffin and the beverage. The test was performed in 120 elderly consumers using a 9-point hedonic scale, ranging from “Like extremely” to “Dislike extremely”. Prior to the test, the participants were informed about the ingredients of each food and were asked about any type of allergy or intolerance they might have to the ingredients. Each food was evaluated separately. Participants were given 10 g or 15 mL of bread or beverage, respectively, at room temperature. Each sample was presented monadically in a plastic cup (2 oz or 1 oz, respectively), labeled with three-digit random numbers. Participants rinsed their mouths with purified water (Alaska**^®^**, Ciudad Juárez, Chihuahua, Mexico) at the beginning of the session. They tasted each sample and used the hedonic scale to indicate the degree the degree to which they liked the sample [16].

### 2.3. Participants of Study

Fifty-eight EI aged 60 years and above (29 men and 29 women) from three nursing homes in Ciudad Juárez, Chihuahua, were evaluated for cognitive capacity (Pfeiffer test) [17] and affective status using the Geriatric Depression Scale [18] for depression and the Golbert Scale [19] for anxiety. EI were informed about the study. They were asked about any liver or kidney dysfunction, and about any type of allergy or intolerance they might have towards some of the ingredients of the foods. Exclusion criteria were considered, such as participants with severe cognitive impairment, a diagnosis of liver or kidney disease, or whose initial biochemical analysis indicated an alteration in these organs. All procedures in the study were approved by UACJ ethical committee (CIBE-2017-1-47 and CIBE-2018-1-37). Twenty-three EI (14 men and 9 women) were eligible, agreed to participate, and completed the study. Informed consent was signed by each participant and a family member. The study consisted of a control stage (C) in which the participants’ usual diet and activities were assessed for 30 days and an intervention stage (I) where the two foods for EI were incorporated into their usual diet. A muffin (51 g) and 100 mL of the beverage (“atole”) were provided at breakfast and dinner for 30 days. The foods were prepared and packaged daily in the Food Sciences Laboratory of UACJ. The foods were provided before the food service in the nursing home and afterward, the EI could intake the foods offered by the respective nursing home. All measurements and analyses of the study were carried out in both stages.

### 2.4. Anthropometric Measurements and Grip Strength

Body weight (BW) (digital scale, SECA**^®^**, Model 874, CDMX, Mexico), knee height, arm, and leg circumference (measuring tape, SECA**^®^**, Model 212, CDMX, Mexico) were recorded in triplicate and body mass index (BMI) was calculated. The height was calculated using Chumlea’s equations for older adults by gender [20]. Furthermore, grip strength was measured by dynamometry (Takei**^®^**, Model 5401, Niigata, Japan). The measurement was carried out in non-consecutive duplicates in both arms. The largest measurement was used.

### 2.5. Body Composition

Body composition (fat-free mass (FFM), fat mass (FM), and total body water (TBW) were measured using electrical bioimpedance (IMPEDIMED**^®^**, Model SFB7, Carlsbad, CA, USA), in accordance with the manufacturer’s instructions.

### 2.6. Sarcopenic Status

The algorithm of the European Working Group on Sarcopenia in Older People (EWGSOP) was used [21]. Additionally, dynapenia was identified in IE, when the strength in women or men was <16 and <27 kg, respectively, in the dominant arm [22].

### 2.7. Nutritional Status

The nutritional status was evaluated with Mini Nutritional Assessment (MNA). This technique is sensitive (96%), specific (98%) and possesses a high predictive value (97%). It classifies older adults as (1) adequate nutritional status (≥24 points); (2) protein-calorie malnutrition (<17 points); and (3) risk of malnutrition (17–23.5 points) [23].

### 2.8. Dietary Intake

A three-day food record (2 weekdays and 1 weekend day) was carried out by trained staff for each participant in both the control and intervention stages. All foods were weighed before being consumed and afterwards, the residue was recorded using a diet scale (TOPCOM**^®^**, Model 200, Taichung, Taiwan). During the EI meals, direct observation was carried out to identify the foods that were not consumed and if the users repeatedly ate certain foods, this was recorded and added to the amount initially served. The food consumption analysis was realized using a food dictionary to express the consumption of macro and micronutrients [24].

### 2.9. Biometric Parameters

#### 2.9.1. Samples

Blood samples were collected from each participant after an 8-hour fast in two different plastic tubes, without anticoagulant and with anticoagulant (SST™, 367986 and K_2_EDTA, 36787861, Vacutainer**^®^**, BD, CDMX, Mexico, respectively). For the general urine test, each participant was asked to collect the first-morning urine sample (using a sterile plastic container 120 mL, TC442, Monterrey, Nuevo León, Mexico), eliminating the first stream of urine. The participants were assisted in this task by nursing home personnel. After collection, the samples were immediately transported to the UACJ Medical Services Laboratory for analysis. After the blood samples were centrifuged (LW Scientific, Model Zip-IQ TT, Lawrenceville, GA, USA) at 3000 rpm for 15 min, and the serum and plasma were separated. The serum was used for blood chemistry tests, while the plasma was placed in conical tubes (Epppendorf**^®^**, Model 3810X, São Paulo, Brazil) and stored at −80 °C for total phenolic compounds and antioxidant capacity tests.

#### 2.9.2. Blood Chemistry Tests

The serum samples were analyzed in the COBAS INTEGRA**^®^** equipment (Roche, Model 400 plus, Sant Cugat del Valles, Spain). Glucose test (GOD-POD enzymatic colorimetric method), triglycerides (GPO-PAP enzymatic colorimetric method), total cholesterol (CHOD-PAP enzymatic colorimetric method), HDL-C and LDL-C (direct method), VLDL-C (calculated from Triglycerides/5), total proteins (cupric ion colorimetric method), albumin (BCG colorimetric method), C-reactive protein (agglutination protein method), creatinine (Jaffé’s kinetic method), urea and blood urea nitrogen (BUN) (indophenol colorimetric method), ureic acid (uricase and peroxidase colorimetric method) were determined. All reactive equipment was purchased from Roche**^®^** (Roche**^®^** México, CDMX, Mexico) and procedures were performed in accordance with the manufacturer’s instructions.

#### 2.9.3. Hematic Biometry

Blood samples with anticoagulant were analyzed in Sysmex**^®^** equipment (Model XS-1000i™, CDMX, Mexico). Erythrocyte quantification, hematocrit, mean corpuscular volume (MCV), mean corpuscular hemoglobin concentration (MCHC), red blood cell distribution (RDW), platelets, neutrophils, lymphocytes, monocytes, eosinophils, basophils, platelets, and platelet volume (hydrodynamic focusing and direct current), hemoglobin (SLS colorimetric method), and leucocytes (semiconductor laser fluorescent flow cytometry method) were determined. All procedures were performed in accordance with the manufacturer’s instructions.

#### 2.9.4. General Urine Test

A physical analysis such as color and appearance was carried out [25]. Density, pH, glucose, ketonic bodies, urobilinogen, bilirubin, leukocyte esterase, nitrites, proteins, and hemoglobin by indirect method (Combur-Test**^®^** strip, Roche México, CDMX, Mexico) were determined using cobas**^®^** urine analyzer (Roche, Model u 411, Sant Cugat del Valles, Spain). All procedures were performed in accordance with the manufacturer’s instructions. Furthemore, microscopic analysis (cells, urinary crystals, mucus, and microorganisms), by a microscopy (Motic**^®^**, Model BA210E, Kowloon, Hong Kong) method, was evaluated.

### 2.10. Phytochemical Characteristics

#### 2.10.1. Total Phenolic Compounds

The extracts from RSF and foods were obtained following a method established by Alvarez-Parrilla et al. [26] with modifications. Briefly, RSF and food samples were dried at 45 °C in a vacuum oven (Shel Lab**^®^**, Model VWR A-143, Tualatin, OR, USA) at 20 mm Hg for 36 h. Next, they were ground using a commercial blender (Nutribullet**^®^**, Model 600w, Capital Brands LLC., Los Angeles, CA, USA) and kept at −18 °C in darkness for no more than 48 h. Next, 5 g of the ground samples were sonicated for 30 min with 10 mL of methanol (JT Baker**^®^**, Fisher Scientific, West Palm Beach, FL, USA) (80%, *v*/*v*), centrifuged at 3500 rpm for 15 min, and the supernatant was collected by filtration, adjusted to a volume of 25 mL, and refrigerated at 4 °C for less than 12 h, until analysis. Total phenolic compounds (TPC) in food extracts and plasma samples were determined by the Folin-Ciocalteu method, according to the methodology described by de la Rosa et al. [27]**,** with slight modifications for the plasma samples. Briefly, 250 µL of the sample was mixed with 1000 µL of sodium carbonate (7.5%) (Merck**^®^**, Toluca, Estado de México, Mexico) and 1250 µL of Folin-Ciocalteu (10% in water) reagent. The mixture was incubated for 15 min at 50 °C and measured at 760 nm by a microplate reader (Bio-RAD**^®^**, Model xMark, CDMX, Mexico). Gallic acid was used as standard, and the results were expressed as milligrams of gallic acid equivalents per 100 g of extract or per milliliter, respectively (GAE/100 g extract or GAE/mL, respectively).

#### 2.10.2. Antioxidant Capacity

The antioxidant capacity (AC) was determined by FRAP (ferric ion reducing antioxidant power) and ABTS (2,2′-azinobis-(3-ethylbenzothiazoline-6-sulfonate) assays for the RSF and designed foods, according to the methodology described by Alvarez-Parrilla et al. [26] and de la Rosa et al. [27]. The AC in the plasma samples was determined by a FRAP assay using FeSO_4_ (JT Baker**^®^**, Fisher Scientific, West Palm Beach, FL, USA) for the standard curves [28]. The FRAP reagent was prepared by mixing 0.3 M acetate buffer (Hycel**^®^**, Zapopan, Jalisco, Mexico) with 10 mM TPTZ (2,4,6-tripyridyl-s-triazine; Acros Organics**^®^**, Morris Plains, NJ, USA) dissolved in 40 mM HCl (Hycel**^®^**, Zapopan, Jalisco, Mexico), and 20 mM FeCl_3_ (Hycel**^®^**, Zapopan, Jalisco, Mexico), in a ratio 10:1:1, *v*/*v*/*v*. The FRAP reagent was heated at 37 °C for 30 min and the assay was performed by mixing 180 µL of the FRAP reagent with 24 µL of the sample in microplate wells. Absorption was measured at 595 nm every 60 s for 30 min in the UV–Vis microplate reader. For the ABTS assay, ABTS radical cation was prepared by diluting ABTS salt (7 mM) (Merck**^®^**, Toluca, Estado de México, Mexico) and K_2_S_2_O_8_ (2.45 mM) (Merck**^®^**, Toluca, Estado de México, Mexico) in phosphate buffered saline (PBS, pH 7.4, 0.15 M KCl) (Merck**^®^**, Toluca, Estado de México, Mexico), and the solution was incubated in refrigeration for 16 h. Then, 12 µL of the sample was mixed with 285 µL of the ABTS radical cation in microplate wells and the absorbance was read at 734 nm for 30 min in the UV–Vis microplate reader. Results were reported in micromoles of Trolox equivalents per 100 g of extract (μmol TE/100 g extract) for RSF and foods and micromoles of Fe^2+^ per milliliter (μmol Fe^2+^/mL) for the plasma samples.

### 2.11. Statistical Analysis

Data from different tests were analyzed by Student’s *t*-test. When the Levene’s test showed significance, a Student’s *t*-test for unequal variances was carried out. Data from the acceptance test were analyzed by a Chi-square test and data from cognitive and affective tests were analyzed using a two-binomial test. All analyses were realized with XLSTAT program version 2019.3.2 (Addinsoft^®^, Paris, France). The results were presented in mean values ± standard deviation (SD). The criterion for statistical significance was *p* < 0.05.

## 3. Results and Discussion

### 3.1. Physicochemical Characteristics, Safety, and Acceptance of Foods for Elderly

Physicochemical analysis of bread (muffin) and beverage (“atole”) designed for EI is shown in Table 1. According to the muffin’s composition and considering a suggested serving of 51 g, the designed bread provides 17% less energy, 3.7 times more protein (9 g), 4.3 times more dietary fiber (2.6 g), 2.8 times less carbohydrates, 5.8 times less sugar (3.1 g), 2.4 times less sodium, and 1.6 times more iron compared with a commercial muffin of the same serving size (vanilla-chocolate cupcake, USDA reference 833282008323 [29]). Once the bread is combined with RSF, it is characterized as a product that is gluten- and lactose-free, has a low content of simple sugars, and a dietary fiber contribution of 10% of the Daily Value (DV).

The beverage (“atole”) designed for EI provides 2 times more protein and 6 times more dietary fiber than a light beverage also combined with RSF for young people [30]. The beverage for EI (100 g portion) compared with other beverages such as 1% low-fat milk (USDA reference 1082), soy milk (USDA reference 16222), almond milk (USDA reference 14091), orange juice (USDA reference 9202), and soft drink (USDA reference 92410310) provides more protein (1.6, 1.5, 10.0, 4.5, and 5.5 times, respectively), dietary fiber (no contribute (NC), 9.6, 9.6, 2.2, and NC times, respectively), Cu (200, 1.9, 9.5, 3.1, and 28.6 times, respectively), Fe (7.0, 1.3, 2.4, 2.1 and 35 times, respectively), K compared with almond milk (5.8 times) or soft drink (35.8 times), and Zn (2 times) compared with orange juice and soft drink. Total sugar content is lower than the 1% low-fat milk (4.9 g/100 g), orange juice (8.6 g/100 g), or soft drink (9.9 g/100 g) [29]. In an 100 g portion, the muffin and beverage contain Cu (20% and 13.3%, respectively) (1.5–3.0 mg DV recommended for EI), Zn (11.3% and 1.3% respectively) (15 mg DV recommended for EI), Fe (55.0% and 7.0%, respectively) (10 mg DV recommended for EI), K (9.2% and 5.1%) (3510 mg DV recommended for adults) and Na (12.3% and 3.9%, respectively) (<2000 mg DV recommended for adults) [29,31,32,33]. In phytochemical characteristics, the results showed that the designed foods had a higher TPC content and a higher antioxidant capacity (AC) than RSF, indicating a possible synergy of the other ingredients. However, the RSF contributed 33.5% of TPC in the muffin and 60.1% in the beverage, while in AC, the RSF contributed to 28.3% of TPC for the muffin and 65.9% for the beverage. Finally, the designed foods demonstrated good properties for the nutrition of the elderly. Microbiological analysis showed that the muffin and the beverage (“atole”) were within the permissible limits, according to the Mexican legislation, in colony-forming units of aerobic mesophilic bacteria (AC) (46 × 10^1^ CFU/g and <1 × 10^1^ CFU/mL, respectively) (Permissible limits: 100 × 10^1^ CFU/g and <1 × 10^1^ CFU/mL, respectively), total coliforms bacteria (CC) (<1 × 10^1^ CFU/g and <1 × 10^1^ CFU/mL, respectively) (Permissible limits: <1 × 10^1^ CFU/g and <1 × 10^1^ CFU/mL), yeast and molds (YM) (<1 × 10^1^ CFU/g and <1 × 10^1^ CFU/mL) (Permissible limits: Not specified) [14,15]. These microorganisms are indicators of preparation conditions and handling of the foods, indicating a possible risk for the presence of pathogenic microorganisms that affect the health of the consumer. The results showed that the muffin and the beverage were safe for consumption by EI [34].

**Figure 1:**
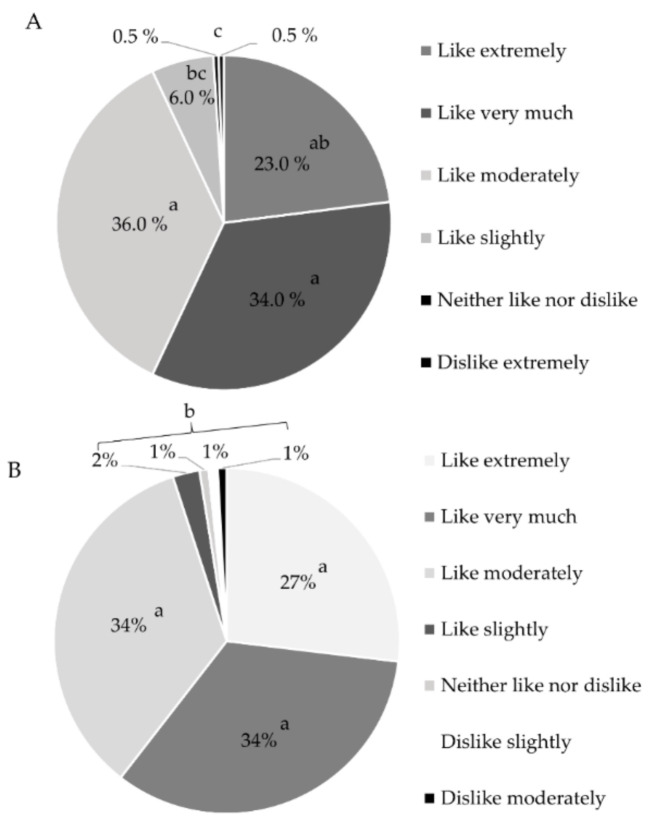
Acceptance of foods designed for the elderly. (**A**). Bread (muffin), (**B**). Beverage (“atole”). 9-point hedonic scale. Different letters indicate significant difference (*p* < 0.05).

The acceptance test for the muffin was carried out in 118 EI in life-free. Participants (32 women and 86 men) were 67.3 ± 7.1 years old. The muffin was accepted by EI in reference to the hedonic scale (7.8 ± 0.9, *p* < 0.01). An analysis by category indicated that the product was within the three highest categories of liking (Figure 1A) for 93% of the IE, only 7 participants classified it as neutral, and one participant indicated a dislike for the bread (*p* <0.01). On other hand, the acceptance test for the beverage (“atole”) was carried out in 119 EI in life-free. Participants (77 women and 42 men) were 67.2 ± 7.4 years old. The beverage was accepted by EI in reference to the hedonic scale (7.9 ± 0.8, *p* < 0.01). An analysis by category indicated that 95% of EI rated the beverage within the three highest categories of liking, and only 5% of the participants indicated a lower level of liking (*p* < 0.01) (Figure 1B).

Feeding the elderly is a challenge that requires special attention, since an adequate diet can help reduce morbidity and quality of life in the elderly. Today, there are some options for functional foods, food supplements, and dietary products, adapted to meet the physiological and nutritional requirements of the elderly. However, these options are limited and, in some countries, inexistent. Functional foods are generally included in EI’s diets, providing nutrients, and preserving the consumer’s state of health. In the European market, examples of this type of food designed for the elderly include cookies with fiber, milk with 3-Omega fatty acids, yogurt with probiotics, among others. In addition, there are also dietary products for the elderly that can be eaten when an additional level of energy and/or nutrients is required. Foods such as breakfast cereals, purees, creams, and instant soups, juices, drinks, and desserts are examples of these dietary products. In some cases, when these options are not available, baby foods such as cereals and purees are used to feed the elderly, however, these products have not been designed to take into account the nutritional requirements of the elderly [35]. In this study, a bread (muffin) and a beverage (“atole”) were designed considering a higher protein intake, dietary fiber, minerals, a reduced intake of sugars. Additionally, these foods were gluten- and lactose-free. RFS was considered a functional ingredient that, in addition to providing nutrients, supplied dietary fiber and secondary metabolites such as phenolic compounds [11,36]. Gluten-free products, such as bread, are characterized by low nutritional properties, poor taste, and inferior quality compared to equivalent products [37]. However, the use of unconventional ingredients has shown the good physicochemical, sensory, and antioxidant properties in bread [38]. In this case, the muffin showed good characteristics and was very well accepted by EI due to its soft and fluffy consistency and its pleasant taste. Furthermore, the beverage (“atole”) presented a thick consistency, a mild odor and a taste of chocolate and cappuccino coffee, which was also well accepted by the EI. When designing foods, it is important to consider the typical ingredients of the population’s diet and their sensory quality in order to make these foods more attractive and thus more likely to be consumed by the elderly [35]. Commonly used textures may not be safe for the elderly, and softer and easier-to-swallow foods are necessary when dysphagia is present. Characteristics such as the taste and acceptance of food encourage older people to chew and swallow them [39].

### 3.2. Functionality of Foods Designed for the Elderly

The participants in this study were 9 women (78.3 ± 5.9 years old) and 14 men (78.3 ± 8.9 years old) living in nursing home conditions. These EI showed mild (*n* = 20) and moderate (*n* = 3) cognitive impairment. In the affective status, 43% of EI maintain interpersonal relationships with family members, especially with grandchildren, while 57% do not. Mild depression was identified in 37% of the participants (23% women and 14% men), and severe depression was observed in 25% of men. Mild anxiety was identified in all of the participants.

#### 3.2.1. Nutritional Indicators of Elderly

The anthropometric characteristics and body composition of the EI by gender at the beginning of the control stage (basal measurements, B) are shown in Table 2. The women were characterized by lower height, total body water, and fat-free mass than the men.

At the end of the control stage (C), the results showed a reduction in the fat-free mass (FFM) of the participants (~2 kg), though no significant changes were identified in the other indicators (Table 3). However, a general deterioration was observed, with a reduction in total body water (TBW) and consequently an increase in fat mass (FM), which is characteristic of this stage of life. Furthermore, a reduction in arm circumference was observed, indicating a decrease in energy reserves. An analysis by gender showed greater fat-free mass (FFM) in men (39.1 ± 6.3 kg) than in women (32.1 ± 6.6 kg) (*p* = 0.02).

On the other hand, the grip strength was greater in men (19.5 ± 6.2 kg) than in women (10.7 ± 5.4 kg) (*p* < 0.01). Sarcopenic status was identified in 17 participants (74%) and 6 EI (26%) showed pre-sarcopenia. Dynapenia was identified in both women (97.5%) and men (93.3%). No significant changes were observed in the cognitive status of EI during the control stage. Furthermore, the MNA test during the control stage (C) showed an adequate nutritional status of EI. However, an analysis by participant showed that 30% of women and 24% of men were at risk of malnutrition. The food intake analysis showed a deficit in energy intake (1547 kcal) (Table 4), corresponding to 94% of the recommended energy consumption (1645 kcal) according to the body weight (BW) of the participants. Adequate distribution of nutrients was observed in carbohydrates (57%), total fat (27%), and proteins (16%) (1.1 g of protein/kg BW/d) during the control stage in the dietary intake of EI. However, an inadequate distribution by type of fat was identified, which was characterized by the consumption of saturated fats, while the monounsaturated and polyunsaturated fatty acids were found to be below dietary recommendations. Cholesterol consumption was greater than 300 mg/d according to the energy intake. In addition, a very low dietary fiber intake (58%) was observed according to the recommendation of 30 g/d for this population [40]. In micronutrients, very low intake of calcium (72% RDI), vitamin C (72% RDI), and very low consumption of zinc (42% RDI), vitamin A (37% RDI), and vitamin E (33% RDI) were also observed [41].

At the end of the intervention stage, when the foods combined with RSF had been consumed for 30 days, the results showed an increase in the BW of the participants (1.1 kg), though no other significant change was identified in the other indicators. FFM and TBW were preserved, as well as energy reserves (arm circumference) and muscle reserves (calf circumference) (Table 3). Results by gender indicated that grip strength was greater in men (19 ± 4.9 kg) than in women (11.7 ± 5.2 kg) (*p* < 0.01). Improved development of EI was observed in the cognitive status from mild deterioration to normal status, but this small change was not significant.

An analysis of the dietary intake after the intervention stage showed nutritional benefits of to the consumption of foods designed for EI and combined with RSF (Table 4). The participants increased their energy intake, reaching 100% of the recommended energy (1645 kcal) for EI. The results indicate an increase in protein intake (29%, 18.6 g) (1.5 g/kg BW/d), dietary fiber (76%, 13.4 g), iron (44%, 4.4 mg), zinc (39%, 1.8 mg), folate (19.6%, 83.4 µg) and a decrease in cholesterol (26.6%, 66 mg) and sodium (15.9%, 319.5 mg). Additionally, there was a reduction in the intake of Omega-6 fatty acids (28%, 1.5 g) and the intake of Omega-3 fatty acids increased by 100% (0.4 g), modifying the ratio of these fatty acids from 13: 1 in the control stage to 5:1 by the end of the intervention stage. The dietary fiber intake reached 100% of the dietary recommendation of 30 g/d [40]. The micronutrient intake increased in iron (100% RDI), zinc (58% RDI), folate (100% RDI), and vitamin A (33.8%, 50% RDI) for this group of elderly individuals. However, a decrease was observed in vitamin C (27.5%, 52% RDI) and vitamin E (43%, 20% RDI) [41].

Elderly individuals experience some disabilities and frailty that cause difficulties in the eating process. These difficulties negatively interfere with the preparation and consumption of food and/or beverages. Eating difficulties are complex and consist of a variety of problems related to the process of eating from the pre-oral phase to the handling of food in the mouth and swallowing, affecting total energy and nutrient consumption. Eating difficulties are a major risk factor for malnutrition among older people [42]. In this study, malnutrition status in EI was higher than that reported in the non-institutionalized elderly [43]. Furthemore, the state of sarcopenia was higher than that reported in institutionalized women in Mexico City [44], which may suggest different associated factors such as diet and lifestyle in the north of the country. Changes in body composition, such as a decline in skeletal muscle tissue and an increase in adipose tissue, are characteristic in the elderly. Many studies have focused on the use of protein and amino acid supplements that, whether combined or not with physical activity, can improve FFM, muscle strength, and functionality in older people, despite the high heterogeneity in the design of the studies [45]. The results of the present work showed a recovery in the body weight of the participants, thus preserving the FFM. This is particularly important given the downward trend in this variable, which has been progressively observed in non-institutionalized older adults despite the maintenance of a balanced diet [46]. This improvement may be related to the small increase in functionality measured through grip strength, which is a marker used for the diagnosis of sarcopenia [22]. Other interventions have tried to improve sarcopenia markers using dairy products [46,47] or oral supplements [48,49], but in this study two foods made with ingredients of plant and animal origin, with an adequate profile of amino acids, minerals, fiber, gluten- and lactose-free, were used. These characteristics of the foods combined with RFS make them optimal products to complement the diet of the institutionalized and non-institutionalized elderly.

In relationship to the energy and nutrients intake by EI, unfortunately few studies describe the intake of the institutionalized population of Mexico. An analysis in the Mexican elderly population indicated an energy intake of 1502 kcal (88% RDI), similar to that identified in this study’s (94% RDI) control stage [50]. However, the consumption of foods added with RSF increased the energy consumed by EI, and protein intake reached 1.5 g/kg BW/d, which has been recently recommended as maintaining muscle mass in older adults [51]. It is important to highlight that protein intake, as well as distribution of protein throughout the day, is important. Recent evidence indicates that breakfast and dinner of foods rich in protein can contribute to the adequacy of this nutrient and healthy aging, especially in the institutionalized elderly population [52,53]. Therefore, it is important that the institutions incorporate a staff training program to monitor the amount, distribution, and time of foods rich in protein, and to identify changes in body weight as an indicator of a change in the body reserves of the elderly. The dietary fiber intake was also increased in the participants of this study. Fiber intake is important from a metabolic point of view due to its influence on lipid and glucose metabolism. Additionally, fiber can act as a prebiotic which reduces incidences of cancer. This effect is very important in the elderly because of the age-related changes in microbiota colonic composition, due to the modification in the gastrointestinal tract, the composition of the diet, and the reactivity of the immune system of the elderly [54]. Recent findings suggest that dietary fiber has a health benefit on skeletal muscle mass in the elderly when an adequate protein intake is consumed and physical activity is performed [55]. The risk of micronutrient deficiencies in the institutionalized elderly population has been studied. Deficiencies in micronutrients are associated with risk of cardiovascular diseases, weight loss, reduction in muscular mass and strength, and a lower tolerance to support chronic or infectious pathological processes [56]. In this study, deficiencies in minerals such as calcium and zinc and vitamins such as C, A, and E, were identified. Consumption of designed foods for the elderly benefitted zinc intake (6.4 mg/d) and strengthened the intake of iron (14.3 mg/d) and folates (508.7 mg/d). Zinc deficiency may be associated with cognitive abilities, mood, and stress in aging. This mineral acts as a neurotransmitter and plays an important role in synaptic function and brain activity [57]. Iron deficiency anemia in older adults causes adverse effects such as a decline in strength and physical activity, cognitive impairment, frailty, and susceptibility to a greater number of comorbidities and mortality; constant monitoring and attention to diet provides better benefits than intensive treatments with supplements [58]. The consumption of folic acid and vitamin B_12_ can reduce hyperhomocysteinemia, which increases with age. It can also reduce the risks or improve the symptoms of cardiovascular or neurodegenerative diseases [59]. However, the diet of the elderly should be revised to increase the consumption of micronutrients such as vitamins C, A, and E, and calcium, which have a high prevalence in the institutionalized elderly population [56]. Finally, the modification of the elderly’s diet with functional foods should be analyzed for the care and prevention of different diseases that affect the elderly.

#### 3.2.2. Biometric Parameters of Elderly

The blood and urine analyses of participants in the control and intervention stages are shown in Table 5. In the control stage, the blood chemistry and hematic biometry parameters were found within the reference values (RV), except the MCHC, which was below the minimum of RV, and the platelet volume, which was above the upper reference limit. Glucose and albumin plasmatic concentration had decreased by the end of the control stage; men experienced the greatest reduction in plasma glucose (B: 93.3 ± 13.6 mg/dL, C: 78.4 ± 4.3 mg/dL) (*p* = 0.04) and albumin (B: 4.5 ± 0.3 g/dL, C: 4.1 ± 0.3 g/dL) (*p* = 0.04). No significant changes were observed in hematic biometry. General urine analysis showed a cloudy appearance in 40% of the participants and very cloudy appearance in 15%. Only one participant (5%) showed a higher concentration of urobilinogen and this same participant was positive for bilirubin in the baseline measurement (B) and at the end of the control stage (C). Some participants (10%) were positive for nitrites, while the presence of proteins was observed at 25 mg/dL (8.7%) and 75 mg/dL (6.5%) in the urine of EI. In the microscopic analysis, leukocytes were observed as limited (43.9%), moderate (26.5%), abundant (9.0%), and uncountable (15.9%) in the urine of the participants; erythrocytes were absent in 72.3%, scarce in 13.7% and uncountable in 6.5% of EI. Bacteria were scarce in 51.4%, moderate in 7.5%, and abundant in 29.2% of the participants. Additionally, mucus filaments were absent in 72.0%, scarce in 11.5%, moderate in 9.0% and abundant in 7.5%; the cylinders were negative for 97.5% and scarce in 2.5% of the samples. The epithelial cells were scarce in 77.0%, moderate in 13.7%, and abundant in 2.2% of EI. Crystals were negative in 87.8%, scarce in 9.7%, and abundant in 2.5% of the participants.

The biometric parameters of EI at the end of the intervention stage were within the RV, except for MCHC, which increased slightly but remained below the lower limit of the reference value. On the other hand, a slight decrease in platelet volume was observed but remained above the upper limit of the reference value. The consumption of foods combined with RSF did not alter the plasma glucose of the participants, and a decrease in total cholesterol was observed, but this change was not significant. However, the analysis by gender indicate that the plasma cholesterol concentration was lower in men (163.2 ± 21.2 mg/dL) compared to women (197 ± 39.8 mg/dL) (*p* = 0.01). The same behavior was observed for triglycerides in men (112.8 ± 37.6 mg/dL) and women (158.5 ± 74.5 mg/dL) (*p* = 0.03).

**Figure 2:**
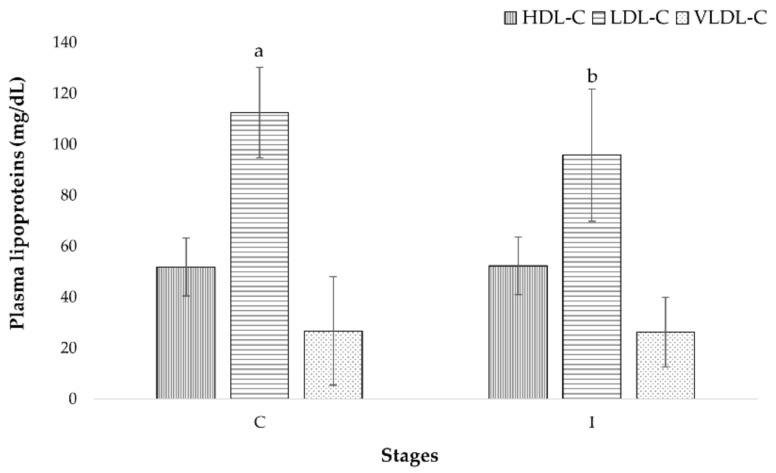
Evolution of plasma lipoproteins of elderly during the study. Mean values ± SD (*n* = 23). C—end of control stage; I—end of intervention stage. Different letters indicate significant differences at *p* < 0.05.

The results showed a decrease in LDL-C lipoprotein (16.7 mg/dL) (Figure 2) and total protein (0.5 g/dL) at the end of this stage of the study. The analysis by gender indicated that men saw a greater reduction in LDL-C (C: 107.1 ± 23.4 mg/dL, I: 84.3 ± 25.5 mg/dL) compared to women (C: 120.9 ± 27.5 mg/dL, I: 113.6 ± 27.9 mg/dL) (*p* = 0.02). Additionally, men showed a greater decrease in total protein (C: 7.3 ± 0.6 g/dL, I: 6.8 ± 0.4 g/dL) (*p* = 0.01) that women (C: 7.4 ± 0.6 g/dL, I: l 7.2 ± 0.7 g/dL).

**Figure 3:**
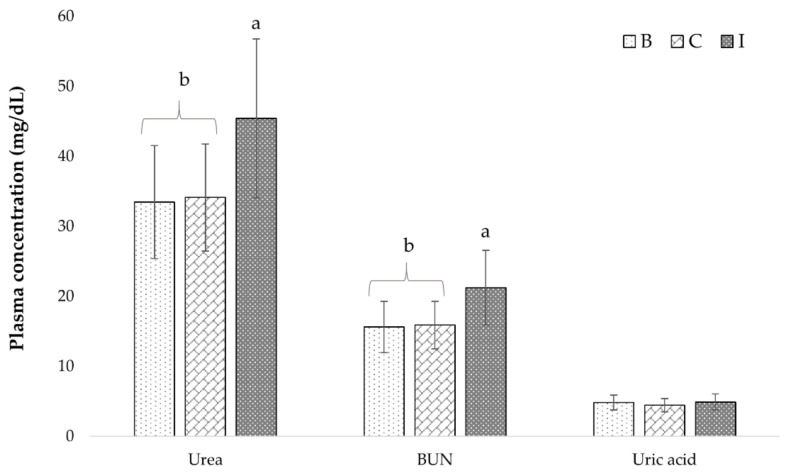
Evolution of urea, BUN, and uric acid in plasma of participants during the study. Mean values ± SD (*n* = 23). B-basal measurements, the start of control stage; C—end of control stage; I—end of intervention stage. Different letters indicate significant differences at *p* < 0.05.

Urea (11.3 mg/dL) and BUN (5.3 mg/dL) increased significantly after food consumption (Figure 3). The analysis by gender indicated that men (Urea: C: 32.1 ± 6.5 mg/dL, I: 41.6 ± 8.4 mg/dL; BUN: C: 14.9 ± 2.8 mg/dL, I: 19.4 ± 3.9 mg/dL) and women (Urea: C: 37.2.1 ± 8.2 mg/dL, I: 51.2.6 ± 12.8 mg/dL; BUN: C: 17.3 ± 3.6 mg/dL, I: 24.0 ± 6.0 mg/dL) increased plasma urea and BUN, with women showing the greatest increase in both parameters (*p* < 0.01).

The general urine examination at the end of the intervention stage did not show significant changes (Table 5). A cloudy appearance was observed in 25.9% of the samples; a very cloudy appearance was observed in 18.7%. Nitrite were positive in 13% of the participants and proteins were identified in 25 mg/dL (8.7%), 75 mg/dL (6.5%) and 100 mg/dL (2.2%) of the EI. In the microscopic analysis, leukocytes were observed as limited (37.0%), moderate (15.2%), abundant (10.9%), and uncountable (17.4%) in the participants’ urine participants; erythrocytes were absent in 64.7%, scarce in 19.6%, moderate in 2.2%, and uncountable in 10.9% of EI. Bacteria were scarce in 56.5%, moderate in 2.2%, and abundant in 37.0% of the participants. Futhermore, mucus filaments were absent in 80.4%, scarce in 10.9%, moderate in 6.5%, and abundant in 2.2%; the cylinders were negative for 100% of the samples. The epithelial cells were scarce in 69.9%, moderate in 10.9%, and abundant in 4.3% of EI. Crystals were negative in 91.3% and scarce in 6.5% of the participants [25].

The relationship between cholesterol and atherosclerosis is characterized by its concentration in the plasma, beginning with an accumulation of cholesterol esters in the arteries and producing a lesion which forms an atherosclerotic plaque that continues to grow over time. The growth of the plaque causes the rupture of vessels with subsequent thrombosis which can present as coronary heart disease, ischemic stroke, or peripheral artery disease. Most cardiovascular diseases and deaths occur in the elderly. Cholesterol is transported in the plasma by lipoproteins, where LDL-C carries most of the plasma cholesterol (60%). This lipoprotein is responsible for depositing the cholesterol within the arteries, starting the atherosclerotic process [60]. The reduction in the plasma of LDL-C has been related to a reduction in the risk of major cardiovascular events. An analysis by Delahoy et al. indicated that for 25 mg/dL reduction in LDL-C, the relative risk for vascular mortality was 0.89, major vascular events 0.86, major coronary events 0.84, and stroke 0.90 [61]. For Vallejo et al., a reduction of 39 mg/dL was associated with a 25% lower incidence of major cardiovascular events [62]. In this work, after the consumption of foods combined with RSF for 30 days, a reduction of 14.8% in LDL-C was observed. Participants in this study went from a high-normal plasma concentration >100 mg/dL (112.5 ± 26 mg/dL) to an optimal concentration <100 mg/dL (95.8 ± 30.1 mg/dL), maintaining the vascular function of the EI and reducing the cardiovascular risk [63]. Functional foods with an LDL-C-lowering effect can improve health and/or reduce the risk of disease. These foods can help reduce LDL-C levels in people who are statin-intolerant or unable to reach their target LDL-C levels. Functional ingredients and supplements such as plant sterols and plant stanols, red yeast rice, soluble fiber soy protein, probiotics, berberine, bergamot, and policosanol have been effective in reducing LCL-C levels [64]. In foods designed for the elderly in this study, a synergy of their functional ingredients such as RSF, soy protein, and oat fiber may be responsible for the LDL-C lowering effect. The isoflavones contained in soy protein have been reported to be effective in reducing LDL-C [65]. Soybean and soy products are well-known sources of isoflavones, interestingly succinic and cinnamic acids have been tentatively identified in RSF. These organic acids are precursors in the biosynthesis of flavonoids, isoflavones, and stilbenes [11]. On the other hand, the designed foods were characterized as a good source of dietary fiber due to the RFS and oat fiber. Soluble fiber from oats has been reported to bind cholesterol and bile acids in the intestinal lumen, making them unavailable for intestinal absorption and increasing their excretion in the feces. This excretion stimulates the synthesis of bile acids and cholesterol in the liver, then the expression of the LDL-C receptor is upregulated, causing a reduction in the concentrations of total cholesterol and LDL-C in the circulation. The β-glucans, found in soluble fiber, at a dose of 3.5 g/day have been shown to modestly reduce LDL-C concentration (4.2%) [64]. In this study, the muffin and the beverage provided 13.8 g/day of dietary fiber in addition to the usual intake of fiber by the EI. The recommendation of dietary fiber intake (30 g/day) was reached and a 16.7 mg/dL (14.8%) reduction in LDL-C was observed. Additionally, β-glucans from the intake of oats (1.1 to 7.6 g/day) showed a total cholesterol reduction of 5.9 mg/dL [66]. In the present study, the total cholesterol of the participants was reduced by 14.5 mg/dL after the intervention stage. Soluble fiber can also be fermented by the colonic microbiota, producing short-chain fatty acids (SCFAs) which are transported to the liver via the portal vein and inhibit the endogenous synthesis of cholesterol [64]. Studies on the characterization of fibers in RSF are necessary to determine its effect on cholesterol and LDL-C in the plasma.

On the other hand, in this study presarcopenia (26%) and sarcopenia (74%) were identified in the participants, with a risk of malnutrition in women and men (30% and 24%, respectively) despite adequate protein intake (1.1 g/kg BW/d). During the intervention stage, protein intake increased to 1.5 g/kg BW/d, where the designed foods provided 29 g of protein/day for 30 days. This higher protein intake was reflected in the plasma concentration of urea and BUN (33%) of the EI. Urea and creatinine are nitrogenous end products of the metabolism. Urea is the main metabolite derivative of dietary protein and tissue protein turnover. The BUN corresponds to the nitrogen of urea. Both indicators vary according to factors such as protein intake, endogenous protein catabolism, hydration status, hepatic urea synthesis, and renal urea excretion [67]. NHANES data showed that protein intake was directly associated with blood urea nitrogen (BUN) concentrations, but those in the highest decile for protein intake (~1.4 g/kg/d) still exhibited normal BUN (14.8 ± 0.3; reference range, 7–20 mg/dL). Consuming high protein diets during energy-deficient and body weight loss helps to preserve muscle mass in an otherwise catabolic physiological environment [68]. The protein intake recommendations for EI between 1.0 and 1.2 g/kg BW/d to maintain and regain muscle, between 1.2 and 1.5 g/kg BW/d for elderly with chronic diseases, and 2.0 g/kg BW/d with marked malnutrition [69]. According to Stout [70], 25 to 30 g of protein/meal is recommended, and essential amino acids (EAA) are important to achieve maximum stimulation of muscle synthesis. Furthermore, the distribution of protein intake throughout the day is essential for the maintenance of muscle mass and function. It is important to consider that RSF is characterized by having an interesting amino acid profile since its proteins contain all EAA [10]. Additionally, soy protein is a complete source of EAA [71]. According to Peña-Ordoñez et al. [72], high-biological value protein intake is a protective factor against sarcopenia since each gram reduces the risk by 3%. Together, these observations show that consumption of foods combined with RSF provided a high-protein intake that maintained the muscular reserve of the EI, which may be related to a lesser advance of the progressive deterioration inherent to age and lifestyle.

#### 3.2.3. Phytochemical Indicators in Plasma of Elderly

**Figure 4:**
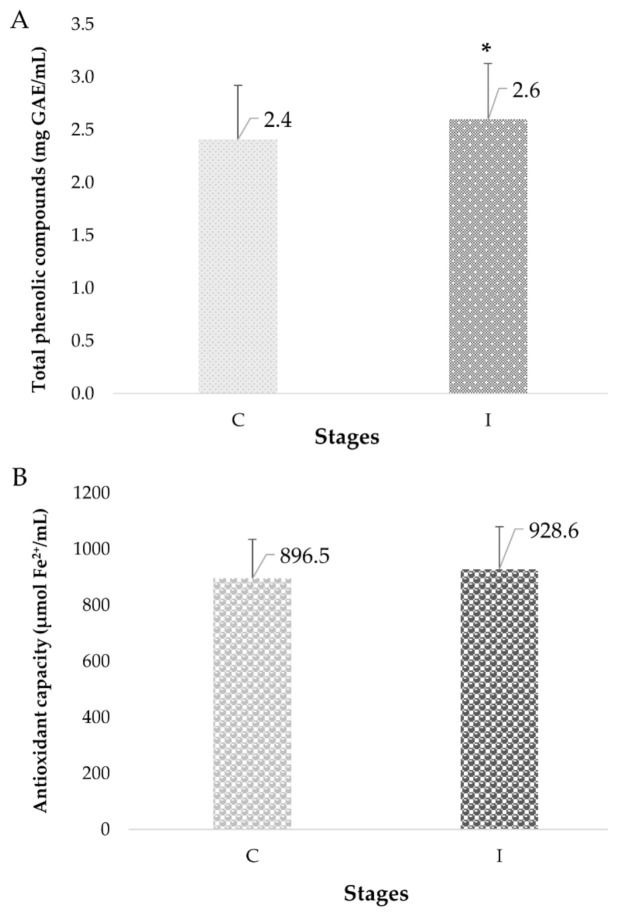
Effect of the consumption of foods added with *Brosimum alicastrum* Sw seed flour on the total phenolics compounds content (**A**) and antioxidant capacity (**B**) in plasma of elderly. Mean values ± SD (*n* = 23). * Indicates significant difference at *p* < 0.05.

The TPC content increased significantly at the end of the intervention compared to the control stage (7.8%) (0.2 mg GAE/mL) (*p* = 0.03) (Figure 4), which indicated a functional property of the foods designed for EI and combined with RSF. An analysis by gender indicated that the TCP content was higher in women (10.7%) than in men (5.8%) (*p* = 0.01). This increase in TPC content was related to an increase of 32.2 μmol Fe^2+^/mL in the AC of the plasma (3.6%), however, this change did not significant. The analysis of AC by gender indicated a higher increase in women (44.0 μmol Fe^2+^/mL, 5%) than in men (24.5 μmol Fe^2+^/mL, 2.7%), which corresponds to the same effect observed in TPC content in women.

The consumption of foods containing RSF had a favorable effect by increasing the plasma concentration of TPC of the IE. Several studies indicate that oxidative stress increases with age and constitutes a factor in neurological damage, dementia, depression, and chronic degenerative diseases such as diabetes, atherosclerosis, and hypertension [2]. A diet rich in antioxidant compounds can help reduce the oxidative stress inherent in aging. Currently, the identification and physiological effect of secondary metabolites from foods of animal and plant origin is a relevant research topic [7]. RFS showed a higher content of TPC and total flavonoids than wheat flour. This content of phenolic compounds was related to a higher antioxidant capacity than wheat flour and a tortilla substituted by 25% of RSF. Furthermore, the TPC content in RFS was higher than that of other seeds such as walnuts, pecan nuts, pistachios, or almonds [11]. In the present study, the foods designed for EI showed a higher TPC content (muffin 2.9 times higher and beverage 1.6 times higher) and antioxidant capacity (muffin from 3.0 to 4.2 times higher and beverage from 1.3 to 1.8 times higher) than the RFS, which may be due to a synergistic effect of the different ingredients in the foods. Twenty phenolic compounds have been tentatively identified in RFS, being mainly phenolic acids esterified with quinic acid as chlorogenic acid and the caffeoyl, coumaryl, and feruloyl derivatives were abundant in RFS. These compounds are typical derivatives of coffee beans and have been linked to cardiovascular health. Resveratrol was also identified in a small amount and some flavonoids such as syringetin, kaempferol and isoquercetin were also identified [11]. Many of the phenolic compounds present in foods are associated with the fiber matrix and synergistically interact with the colonic microbiota by generating active metabolites with antioxidant activity. Low molecular weight polyphenols (from 5 to 10%) are potentially bioavailable to be absorbed through the small intestinal mucosa, producing metabolic and systemic effects [73]. This absorption of TPC was observed in an increase in these compounds in the plasma of EI in this study. Chlorogenic acid, one of the main phenolic compounds in RSF, has been reported as a compound of interest with health-promoting properties such as antioxidant, anti-inflammatory, antilipidemic, antidiabetic, and antihypertensive. Interestingly, a study of rats showed that chlorogenic acid at doses of 10 mg/kg (BW) significantly reduced LDL-C [74]. However, studies on the intake of antioxidant compounds and their effect on the health of elderly people are necessary for a better understanding. Finally, the foods designed for EI and combined with RSF provided phenolic compounds as part of a healthier diet for EI.

## 4. Conclusions

The consumption of bread (muffin) and beverage (“atole”) combined with *Brosimum alicastrum* Sw. (Ramón) seed flour improved the nutritional status of the elderly participants, preserved their energy and muscles reserved and increased their intake of protein, dietary fiber, iron, zinc, folate and reducing the cholesterol and sodium. However, deficiencies in vitamin C, A, and E remained. The ratio of 6-Omeg and 3-Omega fatty acids had improved by the end of the study. The health status of the elderly was favorably impacted after the food intake by reducing LDL-C lipoproteins and increasing the urea concentration and urea nitrogen balance (BUN) in the plasma due to increased protein intake (1.5 g/kg BW/d). The total phenolic compounds were increased in the plasma of the participants, particularly in women. Together with these results, the consumption of these foods, specially designed for the elderly, improves their nutritional status and provides a protective cardiovascular effect, thus improving the health of this vulnerable section of the population.

## 5. Patents

Patent request: MX/a/2019/005290. Formulation and process of dough to obtain bakery products, preferably a muffin, containing *Brosimum alicastrum* Sw. seed flour (Ramón).

Patent request: (In process). Formulation and process of a nutritive beverage type “atole” combined with Ramón seed flour (*Brosimum alicastrum* Sw.).

## Figures and Tables

**Table 1:** Physicochemical composition and phytochemical characteristics of foods added with *Brosimum alicastrum* Sw. seed flour and specially designed for the elderly.

	RSF *	Muffin	Beverage
Energy (kcal/100 g)	336	353	54
Moisture (%)	13.3 ± 0.14	36.3 ± 0.22	81.8 ± 0.06
Protein (%)	11.5 ± 0.39	18.1 ± 0.12	5.5 ± 0.07
Fat (%)	0.6 ± 0.00	25.3 ± 0.24	0.1 ± 0.00
Ashes (%)	3.4 ± 0.11	1.8 ± 0.05	0.7 ± 0.01
Total carbohydrates (%)	71.2 ± 0.56	18.5 ± 0.23	11.9 ± 0.01
Total sugars (%)	NS	6.2 ± 0.20	3.3 ± 0.01
Dietary fiber (%)	13.0 ± 0.21	5.1 ± 0.20	4.3 ± 0.10
pH	5.5 ± 0.01	8.0 ± 0.11	6.3 ± 0.00
Activity water (Aw)	0.30 ± 0.02	0.95 ± 0.00	96.0 ± 0.04
Cu (mg/100 g)	0.5 ± 0.10	0.3 ± 0.00	0.2 ± 0.01
Zn (mg/100g)	1.0 ± 0.10	1.7 ± 0.02	0.2 ± 0.00
Fe (mg/100 g)	4.0 ± 0.70	5.5 ± 0.01	0.7 ± 0.00
K (mg/100 g)	1256.0 ± 12.00	323.7 ± 5.82	178.9 ± 4.51
Na (mg/100 g)	47.0 ± 0.10	245.2 ± 3.96	78.7 ± 4.77
TPC (mg GAE/100 g extract) ^†^	89.8 ± 3.2	268.7 ± 11.8	149.3 ± 6.8
AC (FRAP) (μmol TE/100 g extract) ^†^	209.6 ± 8.1	637.7 ± 36.6	268.3 ± 11.9
AC (ABTS) (μmol TE/100 g extract) ^†^	371.5 ± 23.2	1553.4 ± 75.3	691.2 ± 28.9

Mean values ± SD. RSF-Ramón seed flour; NS-not specified; TPC-total phenolic compounds; AC-antioxidant capacity; EAG-gallic acid equivalents; TE-trolox equivalents. * RSF data from Subiria et al. [11], except data for TPC and AC. ^†^ Data determined in this study. Note: The RFS batch used in Subiria’s study was the same one used in this study to make the muffin and the beverage in this study.

**Table 2:** Anthropometric parameters and body composition of elderly beginning the control stage.

	Men		Women		*p*
	Mean	SD	Mean	SD	
Height (cm)	162.7	5.9	149.7	2.6	0.000
Weight (kg)	59.7	10.8	55.4	10.9	0.171
TBW (L)	29.5	3.9	25.4	2.7	0.013
FFM (kg)	40.3	5.4	34.7	3.7	0.013
FM (kg)	18.5	6.4	17.6	5.3	0.728
BMI (kg/m^2^)	21.4	3.7	23.4	1.8	0.244

Mean values. TBW-total body water; FFM-fat-free mass; FM-fat mass; BMI-body mass index. SD-standard deviation. Significant difference at *p* < 0.05.

**Table 3:** Evolution of anthropometric parameters, body composition, cognitive and nutritional status of elderly before and after consuming foods.

Parameters	B	C	B–C *p*	I	C–I *p*
Body weight (kg)	56.3 ± 9.6	55.9 ± 9.5	0.59	57.0 ± 9.3	<0.01
Arm circumference (cm)	29.0 ± 12.2	26.2 ± 3.1	0.33	26.0 ± 3.2	0.50
Calf circumference (cm)	30.7 ± 2.9	30.6 ± 2.9	0.45	31.0 ± 3.0	0.14
TBW (L)	27.9 ± 4.0	27.0 ± 4.9	0.05	27.1 ± 5.0	0.88
FFM (kg)	38.1 ± 5.5	36.3 ± 7.2	<0.01	37.0 ± 6.8	0.37
FM (kg)	18.1 ± 5.9	18.6 ± 5.7	0.65	20.1 ± 6.3	0.11
BMI (kg/m^2^)	22.1 ± 3.3	22.6 ± 3.6	0.23	22.8 ± 3.4	0.85
Grip strenght (kg)	15.8 ± 6.7	16.3 ± 7.3	0.29	16.5 ± 6.2	0.80
Cognitive assesment	2.5 ± 1.9	2.7 ± 2.3	0.63	2.3 ± 2.6	0.35
MNA	24.2 ± 4.0	24.8 ±3.8	0.19	24.3 ± 3.4	0.44

Mean values ± SD (*n* = 23). TBW-total body water; FFM-fat-free mass; FM-fat mass; BMI-body mass index; MNA—Mini Nutritional Assessment; B—basal measurements, the start of control stage; C—end of control stage; I—end of intervention stage. Significance at *p* < 0.05.

**Table 4:** Energy and nutrients intake for the elderly during the study.

Stage			
	C	I	*p*
Energy (kcal/d)	1547 ± 224	1697 ± 287	0.24
Macronutrients			
Protein (g/d)	63.8 ± 12.6	82.4 ± 14.5	<0.01
Total fat (g/d)	48.0 ± 10.0	54.3 ± 14.4	0.30
Satured fatty acids (g/d)	11.0 ± 1.0	11.8 ± 2.1	0.26
Monounsatured fatty acids (g/d)	15.4 ± 3.0	10.7 ± 3.2	<0.01
Polyunsaturated fatty acids (g/d)	8.9 ± 2.1	6.3 ± 1.8	<0.01
Omega-6 fatty acids (g/d)	5.3 ± 1.6	3.8 ± 1.2	0.01
Omega-3 fatty acids (g)	0.4 ± 0.2	0.8 ± 0.2	<0.01
Carbohydrates (g/d)	222.4 ± 28.8	215.8 ± 33.4	0.66
Total fiber (g/d)	17.6 ± 9.1	31.0 ± 5.9	<0.01
Total cholesterol (mg/d)	320.7 ± 58.5	254.7 ± 85.9	<0.01
Minerals			
Calcium (mg/d)	726.5 ± 178.0	684.3 ± 126.0	0.39
Iron (mg/d)	9.9 ± 2.5	14.3 ± 2.8	<0.01
Potassium (mg/d)	1351.9 ± 467.5	1443.1 ± 290.1	0.34
Sodium (mg/d)	2001.3 ± 545.8	1681.8 ± 588.9	0.03
Zinc (mg/d)	4.6 ± 1.2	6.4 ± 2.4	<0.01
Vitamins			
Vitamin C (mg/d)	60.7 ± 15.1	44.0 ± 13.6	<0.01
Folate (µg/d)	425.3 ± 204.0	508.7 ± 142.5	0.03
Vitamin A (µg RAE/d)	273.8 ± 154.8	366.4 ± 295.6	0.25
Vitamin E (mg/d)	4.4 ± 3.1	2.5 ± 1.6	<0.01

Mean values ± SD (*n* = 23). C—end of control stage; I—end of intervention stage. Significant difference at *p* < 0.05.

**Table 5:** Biometric parameters of elderly during the study.

Parameter	B	C	I	RV
*Blood Chemestry*				
Glucose (mg/dL)	92.0 ± 14.4 ^a^	79.9 ± 7.6 ^bB^	87.2 ± 9.6 ^A^	60.0–100.0
Triglycerides (mg/dL)	119.8 ± 47.4	122.3 ± 43.2	130.7 ± 59.4	45.0–191.0
Total cholesterol (mg/dL)	187.0 ± 21.1	191.0 ± 33.6	176.5 ± 34.2	120.0–200.0
HDL-C (mg/dL)	49.9 ± 11.3	51.8 ± 11.3	52.3 ± 12.5	36.0–76.0
LDL-C (mg/dL)	105.5 ± 17.8	112.5 ± 26.0 ^A^	95.8 ± 30.1 ^B^	80.0–150.0
VLDL-C (mg/dL)	28.5 ± 21.4	26.7 ± 13.6	26.2 ± 11.9	0.0–45.0
Total protein (g/dL)	7.2 ± 0.5	7.4 ± 0.6 ^A^	6.9 ± 0.6 ^B^	6.0–8.5
Albumin (g/dL)	4.4 ± 0.4 ^a^	4.1 ± 0.3 ^b^	4.2 ± 0.3	3.5–5.2
CRP (mg/dL)	0.31 ± 0.3	0.35 ± 0.3	0.40 ± 0.5	0.0–5.0
Creatinine (mg/dL)	0.80 ± 0.1	0.74 ± 0.1	0.83 ± 0.2	0.5–1.5
Urea (mg/dL)	33.4 ± 8.0	34.1 ± 7.6 ^B^	45.4 ± 11.3 ^A^	13.0–60.0
BUN (mg/dL)	15.6 ± 3.7	15.9 ± 3.4 ^B^	21.2 ± 5.3 ^A^	6.0–28.0
Uric acid (mg/dL)	4.8 ± 1.0	4.4 ± 0.9	4.9 ± 1.1	2.7–7.2
*Hematic biometric*				
Erytrocytes (×10^6^/μL)	4.5 ± 0.4	4.7 ± 1.0	4.3 ± 0.5	4.1–5.1
Hemoglobin (g/dL)	14.1 ± 1.3	13.9 ± 1.5	13.5 ± 1.5	12.3–15.3
Hematocrit (%)	43.1 ± 3.7	42.7 ± 4.8	41.2 ± 4.8	36.0–45.0
MCV (fL)	95.3 ± 4.3	95.4 ± 3.8	95.4 ± 3.9	80.0–100.0
MCH (pg)	31.2 ± 1.8	31.0 ± 1.5	32.5 ± 0.8	27.5–32.2
MCHC (g/dL)	32.7 ± 0.9 ^†^	32.5 ± 0.8 ^†^	32.7 ± 0.9 ^†^	33.4–35.5
RDW (%)	14.1 ± 0.7	13.9 ± 0.8	13.9 ± 0.9	11.5–16.0
HD (fL)	48.3 ± 3.3	47.4 ± 2.9	47.1 ± 3.1	0.0–99.9
Lukocytes (×10^3^/μL)	6.4 ± 1.3	6.9 ± 2.3	6.5 ± 1.7	4.5–11.0
Neutrophils (%)	60.1 ± 9.9	61.6 ± 8.2	60.8 ± 7.8	37.0–72.0
Lymphocytes (%)	27.3 ± 8.6	25.9 ± 7.5	26.7 ± 6.9	20.0–50.0
Monocytes (%)	8.6 ± 3.1	8.9 ± 2.7	8.9 ± 2.9	0.0–14.0
Eosinophils (%)	3.6 ± 3.9	3.0 ± 2.2	3.1 ± 2.0	0.0–6.0
Basophils (%)	0.4 ± 0.2	0.4 ± 0.2	0.4 ± 0.2	0.0–1.0
Platelets (×10^3^/μL)	224.6 ± 68.5	236.3 ± 80.0	233.9 ± 72.4	140.0–450.0
Platelet volume (fL)	10.5 ± 1.0 ^†^	11.2 ± 1.3 ^†^	10.7 ± 1.1 ^†^	7.4–10.4
*General urine analisys*				
Colour **	Yellow	Yellow	Yellow	
Appearance **	40% clear	50% clear	61% clear	
Density	1.015 ± 0.007	1.015 ± 0.006	1.017 ± 0.009	1.002–1.030
pH	6.0 ± 1.2	6.3 ± 0.8	5.8 ± 0.9	4.5–8.0
Glucose **	100% negative	100% negative	100% negative	Negative
Ketone bodies **	100% negative	100% negative	100% negative	Negative
Urobilinogen **	95% normal	100% normal	100% normal	0.2 EU/dL
Bilirubin **	95% negative	95% negative	100% negative	Negative
Leucocyte esterase **	65.2% negative	69.6% negative	69.6% negative	Negative
Nitrites **	87.0% negative	91.3% negative	82.6% negative	Negative
Proteins **	87.0% negative	82.6% negative	82.6% negative	Negative
Hemoglobin **	73.9% negative	69.6% negative	69.6% negative	Negative

Mean values ± SD (*n* = 23). B—basal measurements, the start of control stage; C—end of control stage; I—end of intervention stage; RV—reference values from UACJ Medical Services Laboratory. ^†^ Values outside of the RV. Lowercase letters indicate significant differences between B and C measurements, and capital letters indicate significant differences between C and I measurements at *p* < 0.05. Mean values ± SD (*n* = 23). ** Proportion of EI. B–basal measurements, the start of control stage; C–end of control stage; I–end of intervention stage; RV–reference values from UACJ Medical Services Laboratory.

## Data Availability

The datasets generated for this study are available on request to the corresponding author.
